# Eyelid Tumours in Northern Malaysia: A Five-Year Review

**DOI:** 10.7759/cureus.20922

**Published:** 2022-01-04

**Authors:** Tan Qi-Xian, Tan Chew-Ean, Adlina Abdul Rahim, Rona A Nasaruddin

**Affiliations:** 1 Ophthalmology, Hospital Sultanah Bahiyah, Alor Setar, MYS; 2 Ophthalmology, Universiti Kebangsaan Malaysia Medical Centre, Kuala Lumpur, MYS

**Keywords:** five years, eyelid tumours, northern malaysia, histopathological diagnoses, biopsy

## Abstract

Background

The majority of eyelid tumours are benign in nature and constitute 82%-98% of all eyelid tumours. This study aimed to explore the prevalence and frequency of histopathological diagnoses of eyelid tumours encountered in Hospital Sultanah Bahiyah (HSB), Malaysia, from 2016 to 2020.

Method

This study is a case series of 136 patients with eyelid tumours who underwent eyelid biopsy.

Result

Twenty-three (17%) patients were below 18 years old, 64 (47%) patients were between 19 and 64 years old and 49 (36%) patients were more than 65 years old. The mean age in this study was 47.9 ± 25.4 years. The most common benign eyelid tumours were dermoid cyst (31, 22.8%), melanocytic nevus (19, 14%), granuloma (17, 12.5%), squamous cell papilloma (13, 9.6%) and epidermal cyst (12, 8.8%). Most of the benign eyelid tumours occurred at the upper lids (79.8%), and most of the malignant eyelid tumours occurred at the lower lids (81.8%). The most common malignant eyelid tumours were basal cell carcinoma (BCC) (6, 14.3%), followed by malignant lymphoma (3, 6.1%) and sebaceous gland carcinoma (2, 4.1%). Eight out of nine cases of BCC were located at the lower lids. All malignant lymphomas were primary lymphoma. Five were extranodal marginal zone lymphoma of mucosa-associated lymphoid tissue (MALT), and one was follicular lymphoma. All cases with benign and malignant tumours required only a single surgery for excision, and none of the cases had a recurrence.

Conclusion

There were limited references to the epidemiology of histopathological diagnoses of eyelid tumours in Malaysia. Although benign lesions comprise the majority of eyelid tumours, it is essential to delineate between benign and malignant eyelid tumours.

## Introduction

Eyelid tumours occupy a large spectrum of conditions ranging from benign lesions such as dermoid cyst to aggressive, life-threatening malignancy such as sebaceous gland carcinoma [1]. The majority of eyelid tumours are benign in nature and constitute 82%-98% of all eyelid tumours [2]. Understanding the clinical and histopathological characteristics of eyelid tumours can aid in establishing an early diagnosis and hence early treatment for patients.

Hospital Sultanah Bahiyah (HSB) is the tertiary referral centre of oculoplastic service in Northern Malaysia. There is limited data available in the literature that specifically describes the histopathological diagnoses of eyelid tumours in Malaysia. This study aimed to examine the demographic statistic and histopathological diagnoses of eyelid biopsies in a local population.

## Materials and methods

Methodology

This is a case series study.

Inclusion criteria

Patients diagnosed with eyelid tumours and underwent eyelid tumour biopsy at Hospital Sultanah Bahiyah, Alor Setar, Kedah, Malaysia, between January 2016 and December 2020 were included in the study.

Exclusion criteria

Patients who defaulted follow-up during the treatment period, have incomplete clerking history and examination or have eyelid tumours but refused surgery were excluded.

Data collection

The electronic medical records of all 136 patients with the diagnosis of eyelid tumours who underwent eyelid excisional or incisional biopsy in HSB between January 2016 and December 2020 were extracted. Demographic factors (age, gender and race) and clinical data (tumour location and histopathological diagnoses) were collected. The results were analysed using SPSS version 22.0. This study had obtained ethical approval in Malaysia and was conducted according to the principles of the Declaration of Helsinki and obtained local ethical approval (National Medical Research Registry (NMRR) ID-21-02290-28J). Written informed consent was obtained from all the patients prior to eyelid biopsy.

## Results

A total of 136 eyelid tumours that underwent eyelid biopsy were identified during the five-year study period. The mean age of the patients was 47.9 ± 25.4 years. The most common age group were between 19 and 64 years (64, 47%), followed by the age group of more than 65 years (49, 36%) and the age group below 18 years (23, 17%). Females were more affected than males (51.5% versus 48.5%). Most patients (109, 80.1%) were of Malay race. The details of the demographic factors of all eyelid tumours are summarised in Table 1.

**Table 1:** Details of the demographic factors of all eyelid tumours (n = 136)

Demographic factors	Number of patients (n (%))
Age group	
<18	23 (17)
19–64	64 (47)
>65	49 (36)
Gender	
Male	66 (48.5)
Female	70 (51.5)
Race	
Malay	109 (80.1)
Chinese	16 (11.8)
Indian	8 (5.9)
Others	3 (2.2)

Among the 136 patients, 114 (83.8%) eyelid tumours were benign and 22 (16.2%) were malignant. Unilateral eyelid tumours were predominantly encountered (133, 97.8%). Upper eyelid tumours (93, 68.4%) were twice as common as lower lid tumours (41, 30.1%), and only two (1.5%) patients had upper and lower eyelid tumours. The clinical characteristics of eyelid tumours are depicted in Table 2.

**Table 2:** Clinical characteristics of eyelid tumours (n = 136)

Clinical characteristics	Number of patients (n (%))
Types of neoplasm	
Benign	114 (83.8)
Malignant	22 (16.2)
Laterality	
Unilateral	133 (97.8)
Bilateral	3 (2.2)
Location	
Upper	93 (68.4)
Lower	41 (30.1)
Both upper and lower	2 (1.5)

The most common benign eyelid tumours biopsied in HSB were dermoid cyst (31, 22.8%), melanocytic nevus (19, 14%), granuloma (17, 12.5%), squamous cell papilloma (13, 9.6%) and epidermal cyst (12, 8.8%). Among the benign tumours, dermoid cyst demonstrated the youngest mean age of 17 years, while seborrheic keratosis had the oldest mean age of 77 years. Hidrocystoma was more common in females than in males (4:1), and inversely, epidermal cyst was more common in males than in females (0.3:1). Most of the benign eyelid tumours observed typically occurred at the upper lids (79.8%) than at the lower lids (20.2%). One lower eyelid biopsy showed normal tissue in a previously treated squamous cell carcinoma (Table 3).

**Table 3:** Histological classification and frequency of benign eyelid tumours

Types of tumours (n = 114)	Number of cases (n (% of all tumours))	Mean age (n (range))	Sex ratio (n (female:male))	Location (n (upper:lower lid))
Cystic				
Dermoid cyst	31 (22.8)	17 (2–69)	0:2	31:0
Epidermal cyst	12 (8.8)	46 (3–83)	0.3:1	12:0
Melanocytic				
Melanocytic nevus	19 (14)	56 (24–76)	0.5:1	1.1:1
Inflammatory				
Granuloma	17 (12.5)	56 (24–77)	1.1:1	2.4:1
Epidermal				
Squamous cell papilloma	13 (9.6)	56 (19–79)	1.6:1	1.6:1
Seborrheic keratosis	2 (1.5)	77 (77–78)	0:2	2:0
Pleomorphic adenoma	1 (0.7)	28 (28)	0:1	1:0
Lymphoid				
Reactive lymphoid hyperplasia	5 (3.7)	51 (38–66)	0.7:1	0.7:1
Tumours of eccrine and apocrine gland origin				
Hidrocystoma	5 (3.7)	34 (11–66)	4:1	4:1
Vasculogenic				
Capillary haemangioma	2 (1.5)	38 (18–57)	1:1	2:0
Arteriovenous malformation	2 (1.5)	73 (73)	2:0	2:0
Cavernous haemangioma	1 (0.7)	33 (33)	1:0	1:0
Histiocytic				
Xanthogranuloma	1 (0.7)	56 (56)	1:0	0:1
Xanthelasma	1 (0.7)	63 (63)	1:0	1:1
Hair follicle tumour				
Trichilemmoma	1 (0.7)	79 (79)	0:1	1:0
Other				
Normal	1 (0.7)	84 (84)	0:1	0:1

On the contrary, most of the malignant eyelid tumours occurred at the lower lid (81.8%) than at the upper lid (18.2%). Basal cell carcinoma (BCC) accounted for 7.4% of all tumours biopsied and 45.5% of malignant tumours observed in HSB (Table 4). The mean age was 69 years. It was more common in females than in males (1.5:1) and predominantly found at the lower lid (100%). One patient had BCC at the upper and lower eyelid (10%).

Malignant lymphoma was the second commonest malignancy in our study. It accounted for 27.3% of all malignant tumours. The mean age was 62 years and more frequent in males than in females (5:1). All malignant lymphoma cases in our study were located at the lower lids.

**Table 4:** Histological classification and frequency of malignant eyelid tumours

Types of tumours (n = 22)	Number of cases (n (% of all tumours))	Mean age (n (range))	Sex ratio (n (female:male))	Location (n (upper:lower lid))
Haematopoietic				
Malignant lymphoma	6 (4.4)	62 (53–77)	0.2:1	0:5
Plasmacytoma	1 (0.7)	84 (84)	1:0	1:0
Epidermal				
Basal cell carcinoma	10 (7.4)	69 (37–93)	1.5:1	0.1:1
Squamous cell carcinoma	1 (0.7)	84 (84)	0:1	0:1
Sebaceous gland tumour				
Sebaceous cell carcinoma	2 (1.5)	80 (77–83)	1:1	1:1
Melanocytic				
Melanocytic carcinoma	1 (0.7)	82 (82)	0:1	0:1
Tumours of eccrine and apocrine gland origin				
Primary cutaneous mucinous carcinoma	1 (0.7)	82 (82)	0:1	1:0

## Discussion

There were not many epidemiological studies of eyelid tumours published in Malaysia. There are noticeable differences in histopathological diagnoses noted when comparing eyelid tumours at different age groups. The range of the mean age of malignancy cases was between 62 and 84 years old in our study. Malignant eyelid tumours were more common in older age, which concurs with several previous studies [1-6]. This study showed that females were more affected than males (51.5% versus 48.5%), similar to the study of Paul et al. (2011) (53% versus 47%) [6]. Further, Supartoto et al. (2019) also reported a male preponderance (59.6%) in eyelid tumours [7].

Dermoid cyst was the most frequently observed benign eyelid tumours (22.8%), and all were situated at the upper lid in our study. This was followed by melanocytic nevus (14%) as the second commonest benign eyelid tumours. The study of Reddy et al. (1996) of 89 cases of histopathologically proven ocular tumour and tumour-like lesions treated in Kelantan also showed comparable results [3]; the most common benign lesions in that study were dermoid cysts (28.8%), and all were located at the upper lid. Reddy et al. also reported that the second most common benign tumours were melanocytic nevus (8.9%) [3]. Various types of the commonest benign eyelids had been reported in other Southeast Asia countries: nevus (37.7%) and squamous papilloma (15.8%) in Thailand [4], epithelial cyst (7.9%) and dermoid cyst (5.7%) in the Philippines [5], and inflammation (15.9%) and epidermoid cyst (5.3%) in Indonesia [7].

BCC was the commonest eyelid malignancy (7.4%) in our study. This prevalence and incidence rate was almost similar to a previous study (6.7%) [3]. Some previous studies [1,8,9] reported that BCC was the commonest eyelid malignancy in some Western countries, with an incidence rate between 13.1% and 35.6%. On the contrary, of all the eyelid tumours reported, the incidence rate of BCC was lower in Asian countries, ranging from 1.6% to 9.8% [6,10-13]. Few studies reported that sebaceous gland carcinoma was the most common malignant eyelid tumour encountered [14]. The mean age in our study was 69 years old, which is consistent with previous studies that ranges between 54 and 73 years old [1,3,4,5,9-12,14,15]. BCC occurred preferentially at the lower lid in our study with a ratio of 0.1:1 (upper lid/lower lid). This ratio was much lower compared to previously studied ratios (0.3-0.5:1 for upper lid/lower lid ratio) [15]. These are probably due to the increased solar exposure of the lower eyelid. There was no racial distinction among cases reported with basal cell carcinoma in our study.

Out of six malignant lymphoma cases reported in the current study, all were primary tumours and five were extranodal marginal zone lymphoma of mucosa-associated lymphoid tissue (MALT), and one was follicular lymphoma. An equally high incidence rate of malignant lymphoma cases in young to middle-aged adults between 19 and 64 years (6.3%) and older than 65 years was identified (6.1%). It comprised 31.8% of all malignant tumours and 5.1% of all tumours in our study. According to the Malaysia National Cancer Registry Report (MNCR) 2012-2016 published in 2019, lymphoma was the fourth commonest cancer among Malaysian residents, accounting for 5.2%, whereas skin cancer was the ninth most common among Malaysian males (3%) [16]. Approximately 5%-10% of all skin cancers occurred at the eyelids [17]. The incidence rate was higher than the previous study, ranging from 1.4% to 6.5% of all malignant eyelid tumours [5,10,12,14]. The differences in incidence rate mainly are attributed to racial and regional factors.

Sebaceous carcinoma was the third commonest malignant eyelid tumour in our study. There were only two (9%) cases identified. The mean age was 80 years. The incidence rate was much lower compared to studies based on Chinese (38.6%) [18], Indonesian (18.09%) [7] and Indian (31.2%) [19] populations. One patient in our study had regional metastasis to the parotid gland, periparotid lymph nodes and distant metastasis to the anterior chest wall, while another patient had only local involvement at the lower eyelid.

The treatment outcomes of malignant eyelid tumours were also distinctive as compared with benign eyelid tumours. Many patients in our study required a multidisciplinary approach to tumour treatment, especially in malignant tumours, such as requiring pathologist services for histopathology diagnoses, a plastic surgeon for lid reconstruction and haematologist for lymphoma-related oncological treatment.

Upon follow-up, all cases with benign tumours required only a single surgery for excision, and none of the cases had a recurrence. At an average of 10.8 months of follow-up of all 10 BCC cases, none of the cases had reported recurrence. All the cases underwent lid reconstruction with graft or flap comanaged with plastic surgery service. All malignant lymphoma cases were referred to a haematologist. One of the patients had lung metastasis upon follow-up, while the other five cases had only local involvement.

## Conclusions

There were limited references to the epidemiology of histopathological diagnoses of eyelid tumours in Malaysia. This study showed a large spectrum of eyelid tumours reported in Kedah state. Most of the benign eyelid tumours occurred at the upper lids, and most of the malignant eyelid tumours occurred at the lower lids. Although benign lesions comprised the majority of eyelid tumours, demographic and clinical characteristics are also helpful in achieving early diagnosis and appropriate treatment to reduce the morbidity of the disease.

## References

[1] Deprez M, Uffer S (2009). Clinicopathological features of eyelid skin tumors. A retrospective study of 5504 cases and review of literature. Am J Dermatopathol.

[2] Singh U, Kolavali RR (2019). Overview of eyelid tumors. Surgical ophthalmic oncology.

[3] Reddy SC, Das PK (1996). Tumours and tumour-like lesions of the eye: a clinicopathological study from Hospital University Sains Malaysia. Malays J Pathol.

[4] Pornpanich K, Chindasub P (2005). Eyelid tumors in Siriraj Hospital from 2000-2004. J Med Assoc Thai.

[5] Domingo RE, Manganip LE, Castro RM (2015). Tumors of the eye and ocular adnexa at the Philippine Eye Research Institute: a 10-year review. Clin Ophthalmol.

[6] Paul S, Vo DT, Silkiss RZ (2011). Malignant and benign eyelid lesions in San Francisco: study of a diverse urban population. Am J Clin Med.

[7] Supartoto A, Ayuningtyas AN, Dibyasakti BA, Utomo PT, Respatika D, Sasongko MB (2019). The eyelid tumor in Yogyakarta, Indonesia. J Med Sci.

[8] Font RL, Croxatto JO, Rao NA (2006). Tumors of the eyelids. Tumors of the eye and ocular adnexa.

[9] Asproudis I, Sotiropoulos G, Gartzios C (2015). Eyelid tumors at the University Eye Clinic of Ioannina, Greece: a 30-year retrospective study. Middle East Afr J Ophthalmol.

[10] Huang YY, Liang WY, Tsai CC, Kao SC, Yu WK, Kau HC, Liu CJ (2015). Comparison of the clinical characteristics and outcome of benign and malignant eyelid tumors: an analysis of 4521 eyelid tumors in a tertiary medical center. Biomed Res Int.

[11] Gundogan FC, Yolcu U, Tas A (2015). Eyelid tumors: clinical data from an eye center in Ankara, Turkey. Asian Pac J Cancer Prev.

[12] Ho M, Liu DT, Chong KK, Ng HK, Lam DS (2013). Eyelid tumours and pseudotumours in Hong Kong: a ten-year experience. Hong Kong Med J.

[13] Sihota R, Tandon K, Betharia SM, Arora R (1996). Malignant eyelid tumors in an Indian population. Arch Ophthalmol.

[14] Yu SS, Zhao Y, Zhao H, Lin JY, Tang X (2018). A retrospective study of 2228 cases with eyelid tumors. Int J Ophthalmol.

[15] Azizah AM, Hashimah B, Nirmal K (2019). Malaysia National Cancer Registry Report (MNCR) 2012-2016.

[16] Cook BE Jr, Bartley GB (1999). Epidemiologic characteristics and clinical course of patients with malignant eyelid tumors in an incidence cohort in Olmsted County, Minnesota. Ophthalmology.

[17] Xu XL, Li B, Sun XL, Li LQ, Ren RJ, Gao F, Jonas JB (2008). Eyelid neoplasms in the Beijing Tongren Eye Centre between 1997 and 2006. Ophthalmic Surg Lasers Imaging.

[18] Chang CH, Chang SM, Lai YH, Huang J, Su MY, Wang HZ, Liu YT (2003). Eyelid tumors in southern Taiwan: a 5-year survey from a medical university. Kaohsiung J Med Sci.

[19] Kale SM, Patil SB, Khare N, Math M, Jain A, Jaiswal S (2012). Clinicopathological analysis of eyelid malignancies - a review of 85 cases. Indian J Plast Surg.

